# Actin and DNA Protect Histones from Degradation by Bacterial Proteases but Inhibit Their Antimicrobial Activity

**DOI:** 10.3389/fmicb.2016.01248

**Published:** 2016-08-09

**Authors:** Asaf Sol, Yaniv Skvirsky, Edna Blotnick, Gilad Bachrach, Andras Muhlrad

**Affiliations:** ^1^Institute of Dental Sciences, Hebrew University-Hadassah School of Dental MedicineJerusalem, Israel; ^2^Department of Medical Neurobiology, Institute for Medical Research-Israel–Canada, Hebrew University of JerusalemJerusalem, Israel

**Keywords:** histone, DNA, proteases, sepsis, cationic peptides, actins, antimicrobial peptides

## Abstract

Histones are small polycationic proteins located in the cell nucleus. Together, DNA and histones are integral constituents of the nucleosomes. Upon apoptosis, necrosis, and infection – induced cell death, histones are released from the cell. The extracellular histones have strong antimicrobial activity but are also cytotoxic and thought as mediators of cell death in sepsis. The antimicrobial activity of the cationic extracellular histones is inhibited by the polyanionic DNA and F-actin, which also become extracellular upon cell death. DNA and F-actin protect histones from degradation by the proteases of *Pseudomonas aeruginosa* and *Porphyromonas gingivalis*. However, though the integrity of the histones is protected, the activity of histones as antibacterial agents is lost. The inhibition of the histone’s antibacterial activity and their protection from proteolysis by DNA and F-actin indicate a tight electrostatic interaction between the positively charged histones and negatively charged DNA and F-actin, which may have physiological significance in maintaining the equilibrium between the beneficial antimicrobial activity of extracellular histones and their cytotoxic effects.

## Introduction

Histones are highly positively charged polypeptides located in the cell nucleus of eukaryotes. They are the major protein component of chromatin where they spool around DNA and playing an important role in gene regulation (Williamson and Pinto, 2012). There are five major families of histones: H1, H2A, H2B, H3, and H4. While histones H2A, H2B, H3, and H4 are core histones, histone H1 and its H5 derivative are linker histones. The core histones all exist as dimers, they all possess the histone fold domain; three alpha helices linked by two loops. H1, H2A, and H2B histones are rich in lysine and H3 and H4 are rich in arginine (Tagai et al., 2011). Histones are released from neutrophils and from dead cells following apoptosis (Chaput and Zychlinsky, 2009). Upon inflammation, extracellular histones are found in neutrophils extracellular traps (NETs) together with other proteins and DNA (Brinkmann et al., 2004). Histones are separated from DNA and released also during apoptosis (Wu et al., 2002; Wickman et al., 2013) and necrosis (Chen et al., 2014). Histones including H2A (Sathyan et al., 2013), H2B (Robinette et al., 1998; Kawasaki and Iwamuro, 2008; Kawasaki et al., 2008), H4 (Lee et al., 2009), and H1 (Richards et al., 2001) have antimicrobial activity (Katchalski et al., 1952; Hirsch, 1958). They have an important role in protecting skin of vertebrates from bacterial infections. Their smaller derivatives, such as buforin which is a derivative of the H2A histone (Lee et al., 2008; Cho et al., 2009), have higher antimicrobial activity and are essential for protecting the integrity of frog’s stomach endothelium. These small histone-derived peptides also possess anti-endotoxin and anticancer activities (Jang et al., 2011), thus making them attractive reagents for pharmaceutical applications (Cho et al., 2009). However, extracellular histones have also a deleterious side (Xu et al., 2015) since they can act as inflammatory agents synergistically with other secreted compounds (Ginsburg et al., 1993; Koren and Ginsburg, 2015). They may cause several deleterious effects such as endothelial and renal dysfunction, and are major mediators of death in sepsis (Xu et al., 2009). In general, extracellular histones have a significant role in tissue injury and inflammation (Allam et al., 2014).

Actin is a negatively – charged structural protein, which is the most abundant protein in the eukaryotic cell. It has an important role in cytoskeleton formation, cell division, motility, adhesion, signaling, and more (Rottner and Stradal, 2011). Actin exists in either monomer globular (G) or polymer- filament (F) forms, which are interconvertible into each other. Actin interacts with positively charged proteins and peptides, such as lysozyme (Muhlrad et al., 2011) and LL-37 (Sol et al., 2012), which polymerize G-actin to F-actin and bundle F-actin filaments. F-actin protects LL-37 from proteolysis by bacterial proteases and enables its antibacterial activity in their presence (Sol et al., 2014). H1 histone was shown to polymerize G-actin (Magri et al., 1978) and histone H2A–H2B dimer was demonstrated to bundle F-actin (Doyle et al., 2011). Histones bind very tightly to the negatively charged DNA, and form together with DNA highly structured nucleosomes in the cell nucleus. Significant amount of DNA are released from cells following NETosis (Chen et al., 2014) and apoptosis (Williamson and Pinto, 2012). Here we studied the interaction of an extracellular histone mixture with F-actin and DNA. The effect of F-actin and DNA on the antimicrobial activity of the histone mixture and on its proteolysis by bacterial proteases were investigated. We found that both F-actin and DNA protect histone from proteolytic degradation but at a cost of inhibiting the histones antimicrobial action.

## Materials and Methods

### Materials

ATP, ADP, dithiotreitol (DTT), histone type III (histone mixture) and deoxyribonucleic acid (DNA) from calf thymus were purchased from Sigma Chemical Co. (St Louis, MO, USA). Acetone dry powder from back and leg muscles of rabbit was purchased from Pel-freeze Biologicals (Rogers, AR, USA).

### Preparation of Actin

CaATP-*G*-actin was prepared from acetone dried powder derived from the back and leg muscles of rabbit by the method of Spudich and Watt (1971) that even without gel filtration yields highly homogeneous actin in purity greater than 90%. CaATP-G-actin was stored in a buffer containing 5 mM TrisHCl, 0.2 mM CaCl_2_, 0.2 mM ATP, 0.5 mM β-mercaptoethanol, pH 8.0 (CaATP-G-buffer). MgF-actin was polymerized from CaATP-G-actin by 30 min incubation with 2 mM MgCl_2_ at room temperature. MgF-actin was diluted for further treatments in MgF-buffer containing 5 mM MOPS, 2 mM MgCl_2_, 0.2 mM ATP, and 0.5 mM DTT, pH 7.4. The concentration of unlabeled rabbit skeletal muscle CaATP-G-actin and Mg-F-actin was determined spectrophotometrically using the extinction coefficients E1%_290_ = 11.5 cm^-1^. (The optical density of actin was measured in the presence of 0.5 M NaOH, which shifts the maximum of absorbance from 280 to 290 nm). Molecular mass of skeletal actin was assumed to be 42 kDa. Boiled actin: F-actin was heat-denaturated by incubated it in boiling water bath for 10 min.

### Bacterial Strains and Growth Conditions

*Bacillus subtilis* was used as test organism. Tough it is not a pathogen, its relative sensitivity to antimicrobial peptides makes it a preferred model organism to study the antimicrobial actions of antimicrobial peptides (Sol et al., 2014, 2015) and the effect of others molecules on the antimicrobial action of antimicrobial peptides.

*Bacillus subtilis* PY79 (a kind gift of Prof. S. Ben-Yehuda, Hebrew University, Jerusalem, Israel) and *Pseudomonas aeruginosa* PAO1 (from our laboratory stock) were grown in LB Broth (Difco) at 37°C under aerobic conditions. *Porphyromonas gingivalis* ATCC 33277 (from our laboratory stock) was grown in Wilkins Chalgren medium II, (Oxoid, UK) in anaerobic jars (Oxoid) at 37°C. For supernatant collection, overnight cultures of *P. aeruginosa* and 4-day cultures of *P. gingivalis* were centrifuged at 20,000 × *g* for 10 min, and the supernatant was collected and transferred through a 0.2 μm filter (BD Biosciences). Bacterial purity was determined by phase contrast microscopy.

### Growth Inhibition Measurements and Protection Assay

We first tested the antimicrobial activity of 10.5–330 μg/ml histone type III (histone mixture) by dilution of histone in final volume of 50 μl. The reaction mixtures were added to wells of 96-well plates (Nunc, Denmark) containing 150 μl of *B. subtilis* PY79 cells (at mid-logarithmic growth diluted 1:5,000 in LB) to complete a 200 μl final volume. For measuring bacterial growth inhibition activity of histone mixture, the plates were transferred to a GENIOS Microplate Reader (TECAN, Austria), and absorbance at 595nm were measured during incubation at 37°C every 20 min (after automated mixing/aeration for 500 s) to generate growth curves. Percent growth inhibition of each treatment was compared with growth at late logarithmic growth phase of untreated bacteria (0% growth inhibition, ∼10 h). To test the effect of actin and DNA on the growth inhibition action of histones, histone mixture (50 μg/ml) were incubated with or without 50–150 μg/ml F-actin or 10–100 μg/ml DNA for 10 min at 37°C, then, 5 μl of the protease-containing late logarithmic growth supernatant of *P. gingivalis* diluted 1:10 was added (or not) to the 50-μl reaction mixture and incubated for 30 min at 37°C. The reaction mixtures were added to wells of 96-well plates (Nunc, Denmark) containing 150 μl of *B. subtilis* PY79 cells and growth inhibition was measured as described above.

### Proteolytic Digestion and Densitometry

Histone mixture (42 μg/ml or 63 μg/ml) was digested by 4 μl of 10 × diluted *P. gingivalis* or 10 μl of 200 × diluted *P. aeruginosa* supernatant, respectively. Samples incubated in the presence or absence of actin and DNA at 20°C for 30 min. All constituents were added simultaneously, run on 12% SDS PAGE, visualized by Coomassie Blue and evaluated by densitometry. Heat-denaturated *P. gingivalis* and *P. aeruginosa supernatants* were used as controls.

### Statistics

Unless specified, all presented data are mean ± SD of three independent experiments performed in triplicate. All presented SDS gels blots are representative of three independent experiments. Student’s *t*-test was used for calculation of *p* values.

## Results

### Antibacterial Activity of Histone Mixture and Its Inhibition by DNA, F-actin, and Proteolysis

**FIGURE 1:**
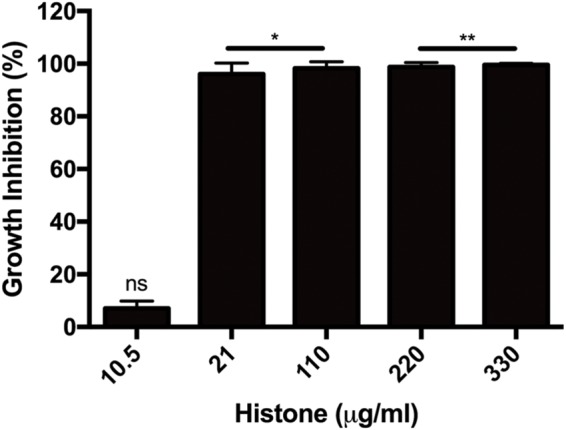
**Growth inhibition of *Bacillus subtilis* caused by 10.5–330 μg/ml histone mixture.** Growth inhibition was evaluated as described in section “Materials and Methods”. The presented data are mean and standard deviation of three independent experiments. ^∗^*p* < 0.05 and ^∗∗^*p* < 0.01.

Several extracellular histones were shown to possess antimicrobial activity. Here we measured the growth inhibition of *B. subtilis* by a histone mixture during 16-h incubation and found histone to be a potent inhibitor since already at 21 μg/ml, bacterial growth was inhibited by 97% after 10 h incubation (**Figure 1**).

**FIGURE 2:**
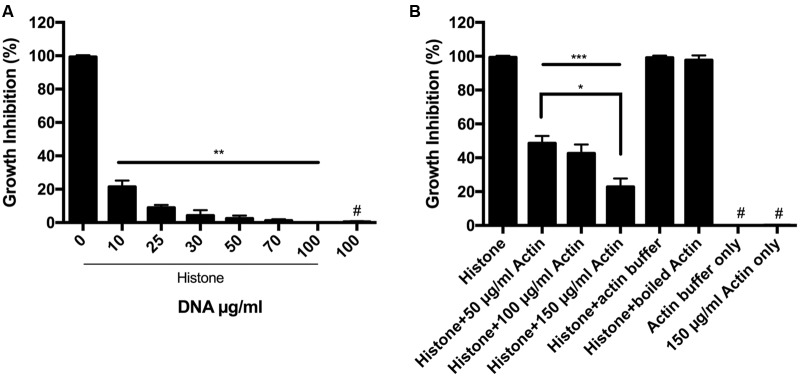
**Antimicrobial activity of histone in the presence of DNA and F-actin.** **(A)** Effect of 10–100 μg/ml DNA **(A)** or 50–150 μg/ml F-actin **(B)** on the growth inhibition of *B. subtilis* caused by 50 μg/ml histone mixture. Growth inhibition was measured as given in section “Materials and Methods”. The presented data are mean and standard deviation of three independent experiments. ^∗^*p* < 0.05, ^∗∗^*p* < 0.01, and ^∗∗∗^*p* < 0.005. # indicated no growth inhibition observed.

It is well known that DNA forms a very tight structured complex with histones in the cell nucleus. We, therefore, studied how purified DNA affects the antimicrobial activity of extracellular histone mixture (**Figure 2A**). We found that DNA abolishes the growth inhibition activity of the histone mixture in a concentration dependent manner. Moreover, the antimicrobial activity of a 50 μg/ml histone mixture decreased by 80% in the presence of 10 μg/ml DNA and was completely abolished by 100 μg/ml DNA present during overnight incubation.

It was previously suggested that H1 could polymerize G-actin (Magri et al., 1978) and that histone H2A–H2B dimer can mediate the bundling of F-actin (Doyle et al., 2011) demonstrating that the negatively charged actin interacts with the positively charged histones. We studied the effect of F-actin on the antimicrobial activity of histone mixture during overnight incubation (**Figure 2B**). 50–150 μg/ml F-actin was found to decrease growth inhibition of *B. subtilis* challenged with a 50 μg/ml histone mixture by approximately 55–80%, respectively. Actin buffer or 150 μg/ml F-actin alone without histone did not affect the growth of *B. subtilis* (**Figure 2B**).

**FIGURE 3:**
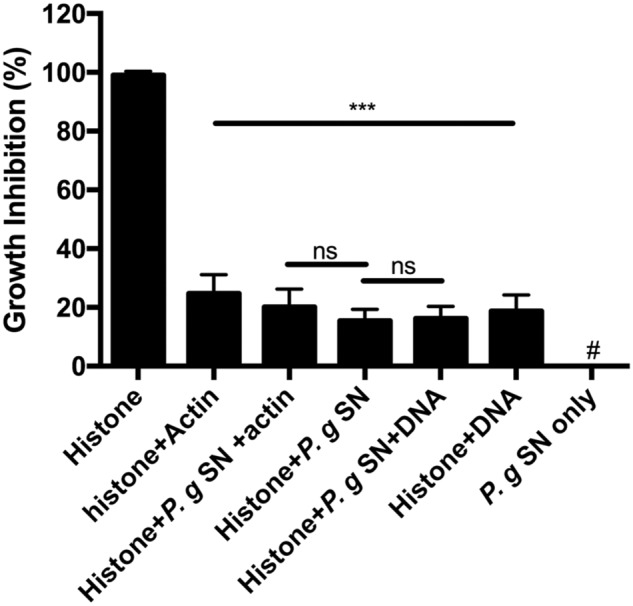
**Effect of 150 μg/ml F-actin, 15 μg/ml DNA and digestion by 1:10 diluted *Porphyromonas gingivalis* supernatant (P.g. SN) on the growth inhibition of *B. subtilis* caused by 50 μg/ml histone mixture.** Growth inhibition was measured as given in section “Materials and Methods”. The presented data are mean and standard deviation of three independent experiments. ^∗∗∗^*p* < 0.005. # indicated no growth inhibition observed.

**FIGURE 4:**
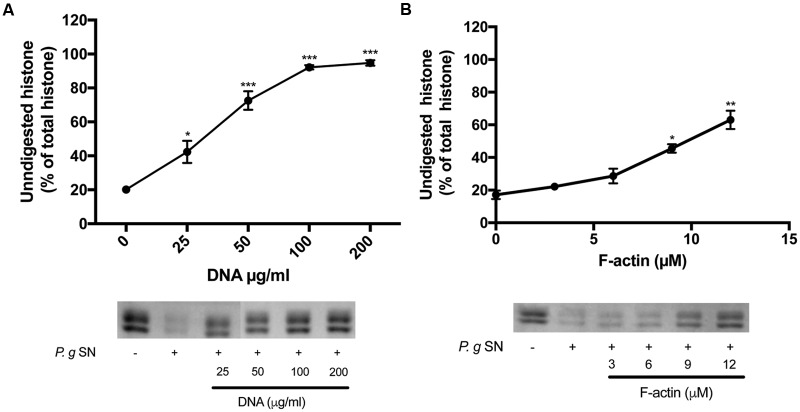
**Effect of DNA and F-actin on the digestion of histone mixture by *P. gingivalis* proteases.** Histone mixture (42 μg/ml) was digested by 4 μl of P. g SN diluted 1:10 in the presence or absence of 0–200 μg/ml DNA **(A)** or 0–12 μM F-actin **(B)**. The effect of DNA and F-actin on histone digestion was measured by calculating the undigested histone proportion in SDS-PAGE. Digestions were carried out at 20°C for 30 min, samples were run on SDS-PAGE and evaluated as described in section “Materials and Methods”. The presented data are mean and standard deviation of three independent experiments. ^∗^*p* < 0.05, ^∗∗^*p* < 0.01, and ^∗∗∗^*p* < 0.005.

The antimicrobial activity of the histone mixture was inhibited by the supernatant of *P. gingivalis* (**Figure 3**). Upon addition of bacterial supernatant, the antimicrobial activity of 50 μg/ml histone mixture decreased by 85%. This decrease is due to the proteolytic activity in the supernatant as shown by **Figure 4**. Unlike the cationic antimicrobial peptide LL-37 that was protected by actin from degradation with supernatant of *P. gingivalis* while retaining its antimicrobial activity (Sol et al., 2014), the presence of 150 μg/ml (3.6 μM) F-actin or 15 μg/ml DNA during the proteolysis did not enable the antimicrobial activity of the histone mixture. The growth-inhibition obtained in the presence of *P. gingivalis* supernatant and DNA or F-actin was very similar to the values obtained in the presence of DNA or F-actin alone.

### DNA and F-actin Protect Histone Mixture from Proteolysis by Bacterial Proteases

As was showed above, both DNA and F-actin inhibit the antimicrobial activity of histones, which demonstrates their interactions with histones. Therefore, we wanted to determine whether the presence of DNA and F-actin can inhibit the proteolysis of histone mixture by proteolytic bacteria.

**FIGURE 5:**
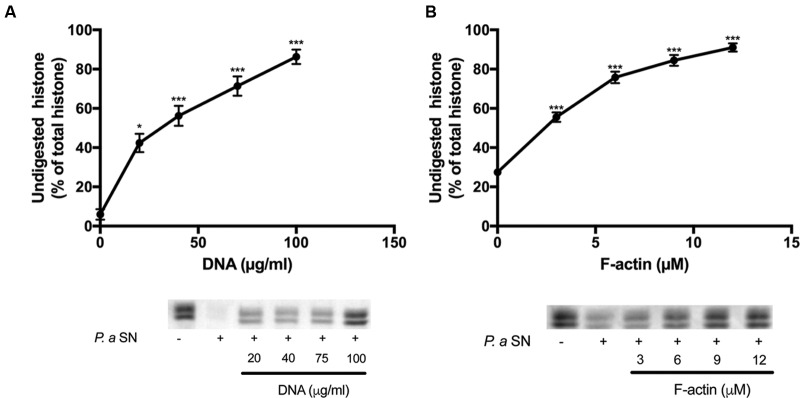
**Effect of DNA and F-actin on the digestion of histone mixture by *P. aeruginosa* proteases.** Histone mixture (63 μg/ml) was digested by 10 μl of 1:200 diluted *P. aeruginosa* supernatant in the presence or absence of 0–100 μg/ml DNA **(A)** or 0–12 μM F-actin **(B)**. The effect of DNA and F-actin on histone digestion was measured by calculating the undigested histone proportion in SDS-PAGE. Digestions were carried out at 20°C for 30 min. Samples were run on SDS-PAGE and evaluated as described in section “Materials and Methods”. The presented data are mean and standard deviation of three independent experiments. ^∗^*p* < 0.05 and ^∗∗∗^*p* < 0.005.

The supernatants of two pathogenic proteolytic bacteria, *P. gingivalis* and *P. aeruginosa* were used as protease sources for studying the proteolysis of the histone mixture (**Figures 4** and **5**). Histone proteolysis by the *P. aeruginosa* supernatant was much stronger than that by *P. gingivalis* as it was possible to dilute the former 200 times and still obtain about 90% digestion of the histone mixture while for *P. gingivalis* supernatant could be diluted only 10 times for the similar degree of digestion. Control heat inactivated *P. gingivalis* and *P. aeruginosa* supernatants did not digest histone mixture. Next, we attempted to digest 42 μg/ml of histone mixture by 10X diluted *P. gingivalis* supernatant in the absence or presence of 0–200 μg/ml DNA (**Figure 4A**), and 0–12 μM F-actin, which corresponds to 126–504 μg/ml F-actin (**Figure 4B**). Both DNA and F-actin protected the histone mixture from proteolysis. However, DNA was a more efficient inhibitor, since 100 μg/ml DNA protected up to 90% of histone from degradation, while 504 μg/ml F-actin protected only up to 65% of the histone mixture from degradation. The digestion of 63 μg/ml histone mixture by *P. aeruginosa* supernatant was carried out under similar conditions as by *P. gingivalis.* Both DNA and F-actin significantly protected the histone mixture from proteolysis by *P. aeruginosa*. 100 μg/ml DNA (**Figure 5A**) or 504 μg/ml F-actin (**Figure 5B**) were needed for decrease the amount of digested histone mixture to ∼10% of the total histone mixture present.

**FIGURE 6:**
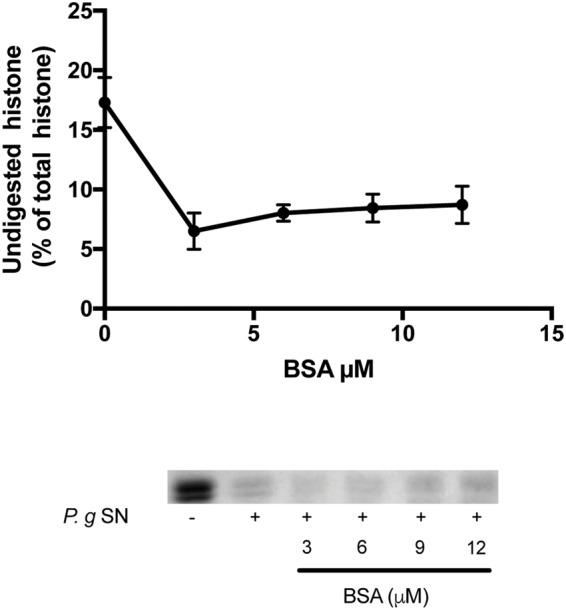
**Effect of bovine serum albumin (BSA) on the digestion of histone mixture by *P. gingivalis* proteases.** Histone mixture (42 μg/ml) was digested by 4 μl of 1:10 diluted *P. gingivalis* supernatant in the presence or absence of 0–12 μM BSA. The effect of BSA on histone digestion was measured by calculating the undigested histone proportion in SDS-PAGE. Digestions were carried out at 20°C for 30 min, samples were run on SDS-PAGE and evaluated as described in section “Materials and Methods”. The presented data are mean and standard deviation of three independent experiments.

Finally, to see the specificity of the ability of F-actin to protect histone mixture against proteolysis, the effect of another protein, bovine serum albumin (BSA), on the digestion of histone mixture was studied (**Figure 6**). In this experiment 42 μg/ml histone mixture was digested by 10X diluted *P. gingivalis* supernatant in the presence of 0–12 μM (0–780 μg/ml) BSA. We found that the presence of BSA does not inhibit but accelerates the digestion of histone mixture. The results indicate that the proteolysis of a histone mixture by bacterial proteases is specifically protected by DNA and F-actin.

## Discussion

Histones are released from activated immune cells (e.g., neutrophils and mast cells) into extracellular traps (NETs) (Brinkmann et al., 2004; von Kockritz-Blickwede et al., 2008) in response to microbial infection. NETs are networks of extracellular fibers composed of DNA, core histones and other antimicrobial factors, which capture and inactivate invading microorganisms (Chen et al., 2014). In addition to NETosis-mediated histone release, apoptotic or necrotic cells can also release histones (Wickman et al., 2013). Histones possess a strong antimicrobial activity at surprisingly low concentrations (**Figure 1**), which protects the organism against invading microorganisms. However, release of large amounts of histone due to a severe bacterial infection can have dangerous deleterious effects on host since extracellular histones are cytotoxic (Ginsburg et al., 1993) and mediate a systemic inflammatory response syndrome including life threatening sepsis (Xu et al., 2009).

Bacteria protect themselves against the strong antimicrobial activity of host defense peptides and histones using proteases, which degrade these protein-based immune factors. Polyanions, which are bound to polycationic histones (Ginsburg et al., 1993) may protect extracellular histones against the bacterial proteases. We found that polyanionic DNA, a very significant constituent of NETs, and F-actin, which is released into the extracellular space upon necrosis and cell death (Sol et al., 2014), might bind histones and inhibit their digestion by bacterial proteases (**Figures 4** and **5**). However, unlike the actin protection of the LL-37 antimicrobial peptide from bacterial degradation, the protection of histones against bacterial proteases by DNA and F-actin does not enable the antimicrobial action of histones. This because DNA and F-actin themselves strongly inhibit the bactericidal activity of histones (**Figure 2**).

The inhibition of the antimicrobial action of extracellular histones by DNA and F-actin has a physiological significance since the large amounts of histones released from the cells upon acute inflammation in addition to their beneficial bactericidal effect, could endanger the host because of their cytotoxicity. It seems that the proper equilibrium between the beneficial antimicrobial and the deleterious cytotoxic effects of extracellular histones, by their release in NETosis, apoptosis or from necrotic cells in on one side, and the inhibition of their action by DNA and F-actin and polyanions in general on the other side, is important for the host’s health. The change of this balance by the sudden release of great amounts of histones without the release of adequate amount DNA and actin could lead to serious pathological consequences such as tissue injury and inflammation (Allam et al., 2014) and the development of fatal sepsis.

Bacteria have DNA binding proteins that resemble histones (DNABII). These proteins which include the integration host factor (IHF) have gene-expression regulatory functions but also an extracellular important role in biofilm formation. Through interactions with extracellular DNA they form a network that is part of the biofilm matrix (Brockson et al., 2014). Among the bacteria that are dependent on IHF for biofilm formation is *P. aeruginosa.* Antibodies that block IHF, greatly impaired biofilm development by *P. aeruginosa* (Estelles et al., 2016). Being proteolytic bacteria which degrades histone (**Figure 5**), the question arises how IHF is not cleaved by the proteases of *P. aeruginosa*? Our results suggest that DNA might protect IHF from self-cleavage by *P. aeruginosa.*

## Author Contributions

AS, designed and carried out experiments, participated in writing the ms; YS, carried out experiments; EB, carried out experiments; GB, designed experiments, and participated in writing ms; AM, designed and carried out experiments and participated in writing ms.

## Conflict of Interest Statement

The authors declare that the research was conducted in the absence of any commercial or financial relationships that could be construed as a potential conflict of interest.
